# Climate‐smart agriculture adoption intensity in a semi‐arid agricultural landscape: drivers and disparities

**DOI:** 10.1002/jsfa.70920

**Published:** 2026-07-27

**Authors:** Mozhgan Nozari, Ali Firoozzare, Flavio Boccia, Sima Ghazanfari, Daniela Covino

**Affiliations:** ^1^ Ferdowsi University of Mashhad Mashhad Iran; ^2^ Department of Economic and Legal Studies University of Naples Parthenope Naples Campania Italy

**Keywords:** farmer behavior, generalized Poisson model, under‐dispersed, climate‐smart agriculture (CSA), Mashhad

## Abstract

**Background:**

Climate‐smart agriculture (CSA) has emerged as a key strategy for enhancing agricultural productivity, strengthening resilience to climate change, and reducing environmental impacts. Understanding the factors that influence farmers' adoption of CSA practices (CSAPs) is essential for designing effective policies and support mechanisms. This study examined the adoption of CSAPs among farmers and identified the socioeconomic, institutional, and behavioral factors affecting their utilization. Data were collected through a structured questionnaire administered to 430 farmers in a major agricultural region of Iran. The determinants of CSA adoption were analyzed using a generalized Poisson model, which is particularly suitable for under‐dispersed count data.

**Results:**

The results revealed that education, self‐efficacy, membership in agricultural cooperatives, access to extension services, availability of credit facilities, job satisfaction, trust in innovative agricultural inputs, and crop insurance significantly increased the likelihood of CSA adoption. Conversely, limited access to training programs and inadequately aligned government support negatively affected adoption levels. A significant difference also emerged between urban and rural farmers, with rural farmers demonstrating a greater propensity to adopt CSAPs.

**Conclusion:**

The results highlight the importance of strengthening farmers' capacities through education, extension services, financial support, and risk‐management tools. Enhancing institutional support, expanding access to training, and promoting trust in agricultural innovations can facilitate broader CSA adoption. These results provide valuable evidence for policymakers and development practitioners seeking to accelerate the transition toward more resilient and sustainable agricultural systems. © 2026 The Author(s). *Journal of the Science of Food and Agriculture* published by John Wiley & Sons Ltd on behalf of Society of Chemical Industry.

## INTRODUCTION

Climate change is recognized as one of the most significant environmental challenges of the current century, strongly affecting the sustainability of natural processes and human activities, particularly agriculture.^1^, ^2^, ^3^ Rising greenhouse gas emissions, altered precipitation patterns, and increasing frequency and intensity of extreme climate events over recent decades have affected crop production and have exerted additional pressure on food security, water and soil resources, and the economic stability of rural communities. These conditions have led to reduced productivity, unstable production, and threats to farmers' livelihoods, positioning agriculture as one of the sectors most vulnerable to climate change.^4^, ^5^ Consequently, escalating environmental, demographic, and economic pressures highlight the urgent need for innovative approaches in agricultural management.

In response to these challenges, climate‐smart agriculture (CSA) has emerged as a comprehensive and innovative approach designed to redesign agricultural systems to cope with climate change.^6^, ^7^ It focuses on three main objectives – sustainably increasing farmers' productivity and income, enhancing adaptability and resilience to climate change, and reducing greenhouse gas emissions – and has attracted considerable attention at national and international levels.^5^, ^8^ It encompasses a dynamic set of technological, managerial, and institutional innovations that can be tailored to local conditions to improve the performance and resilience of farming systems.

International studies have shown that implementing CSA practices (CSAPs) can increase farmers' productivity, strengthen local communities' resilience to droughts and floods, and improve food security.^9^, ^10^, ^11^ Nevertheless, CSA adoption remains low in many developing countries due to factors such as high input costs, limited financial resources, and weak institutional support.^12^, ^13^, ^14^

Research in Iran has identified that awareness of climate change, participation in training and extension programs, access to climate information, and belief in the necessity of climate‐smart technologies are among the key drivers of CSA adoption among farmers. Strengthening social networks, enhancing technical training, and providing institutional and governmental support can also significantly facilitate CSA uptake and promote the transition toward sustainable and resilient agriculture.^15^, ^16^, ^17^, ^18^, ^19^

Focusing solely on the adoption or non‐adoption of individual practices does not capture the complexity of farmers' actual behavior, however. Field evidence indicates that farmers often adopt multiple practices simultaneously or gradually. The number of practices chosen reflects farmers' awareness, access to resources, attitudes toward climate change, and the level of institutional and social support. Analyzing the factors influencing the number of CSAPs adopted therefore provides not only a more realistic and precise understanding of farmers' adaptive capacity but also a practical basis for policy‐making and the design of supportive interventions.^5^, ^12^, ^19^, ^20^

In Iran, particularly in arid and semi‐arid regions, the need to adopt climate‐resilient approaches has become increasingly critical, as the agricultural sector is very vulnerable to climate change. Farmers are compelled to simultaneously employ multiple CSAPs to sustain livelihoods and maintain production stability. Recurrent droughts, the continuous decline of groundwater levels, shifts in precipitation patterns, and rising average temperatures are among the major threats to farmers' livelihoods and sustainable food access.^21^

Razavi Khorasan province, with approximately one million ha under cultivation and ranking first to third nationally in the production of certain strategic crops, is recognized as Iran's third agricultural hub. Within this context, Mashhad, with over 56 000 ha of cultivated land, stands out as one of the main centers of agricultural production in the province. The substantial decrease in rainfall and the rising temperatures in recent years have highlighted the necessity of adopting – and particularly diversifying – CSAPs as an effective approach to reduce vulnerability and enhance the resilience of agricultural systems.^22^, ^23^

Accordingly, the present study aimed to investigate the factors influencing the number of CSAPs adopted by farmers in Mashhad. Understanding these factors provides deeper insights into farmers' adaptation strategies under complex climatic conditions and can inform better management of limited natural resources. The results offer a comprehensive view of farmers' responses to climate change, forming a foundation for policies that safeguard livelihoods, enhance food security, prevent resource degradation, and promote sustainable agriculture while reducing community vulnerability. This research also provides an opportunity to operationalize CSA approaches more effectively within regional agricultural policies, contributing to the achievement of the United Nations Sustainable Development Goals in the agricultural sector.

## MATERIALS AND METHODS

### Study area

Mashhad city is located in northeastern Iran and is the capital of Razavi Khorasan province. Mashhad county covers an area of 9169 km^2^ and is the second largest and most populous county in the country. The region is considered one of Iran's major economic, cultural, and religious hubs. Situated at an elevation of 950–1150 m above sea level, Mashhad county experiences a semi‐arid climate characterized by cold and relatively humid winters and hot, dry summers. During the 2024–2025 agricultural year, the recorded precipitation there was 152 mm, representing a significant decrease of 148 mm compared with the previous agricultural year, which recorded 300 mm of rainfall. The mean temperature for the same period was 16.5 °C.^23^, ^24^

### Data collection

The data for this study were collected through a questionnaire during the 2024 agricultural year. A two‐stage cluster sampling method was employed. In the first stage, several villages were randomly selected from a list of villages in Mashhad. In the second stage, a list of farmers residing in each selected village was obtained through village councils. Respondents were then selected using simple random sampling within each village. The initial sample size was estimated at 385 participants, considering a 95% confidence level and a 5% margin of error. The initial sample size was estimated at 385 farmers based on a 95% confidence level and a 5% margin of error. To increase the robustness of the empirical analysis, data were ultimately collected from 430 farmers using the same two‐stage cluster sampling procedure. All questionnaires included in the analysis were fully completed and met the study requirements.

Table 1 provides a summary of the research characteristics. The information presented in this table offers a comprehensive overview of the study's methodological framework and provides a clearer understanding of the data collection process and the sampling structure.

**Table 1:** Study characteristics

Section	Details
Study area	Mashhad county
Data collection year	2024
Type of research	Applied research
Research method	Survey
Data collection tools	Questionnaire, field survey
Statistical population	Farmers of Mashhad
Sampling method	Two‐stage cluster sampling
Sampling stages	Stage 1: Villages randomly selected; Stage 2: Farmers randomly selected within selected villages
Data source	Village councils
Confidence level and margin of error	95% and 5%
Final sample size	Valid responses 430

### Count models

Count models are important statistical tools used to analyze dependent variables that represent counts – such as the number of occurrences, the frequency of an event, or the count of specific units within a given time or spatial interval. These models allow researchers to examine the relationship between independent variables and a count‐dependent variable, and to assess the effects of various factors on event frequency.

The application of count models is widespread across multiple fields, including economics, social sciences, public health, agriculture, environmental studies, and criminology.^25^, ^26^ One of the most fundamental and widely used count models is the Poisson regression model. This model is based on the Poisson distribution, which assumes that the mean and variance of the dependent variable are equal.^27^, ^28^, ^29^ Within this framework, the response variable represents the number of occurrences of an event within a specific time or spatial interval, and its relationship with explanatory variables is modeled through a logarithmic link function. The advantage of using this link function is that the predicted values always remain non‐negative.^30^

Various types of count models can be applied depending on data characteristics, as discussed in the following section.

#### Poisson regression model

If *X* represents the number of events and *μ* is the mean number of occurrences in a specified time or spatial interval, the Poisson probability function is defined as:

(1) $$P\;\left(X=x\right)=\;\frac{e^{-\mu }\mu^{x}}{x!}\;\text{For}\;x=0,1,2,\ldots$$

##### Key feature

The Poisson distribution assumes equality of the mean and variance: *E* (*X*) = VAR (*X*) = *μ*. This property forms the basis of the Poisson model and allows it to model the distribution of rare events over time or space.

##### Violation of the mean–variance equality assumption

In real‐world data, the mean–variance equality assumption is often violated, leading to two possible situations:

- Over‐dispersion. This occurs when the variance exceeds the mean: Var(*Y*∣*X*) > *E*(*Y*∣*X*). This situation typically leads to underestimation of standard errors, overly narrow confidence intervals, and ultimately, incorrect significance tests, which increase the likelihood of wrongly rejecting the null hypothesis.
- Under‐dispersion. This occurs when the variance is smaller than the mean: Var(*Y*|*X*) < *E*(*Y*|*X*). In this case, standard errors are usually overestimated, test power is reduced, and true cases of statistical significance may not be identified.^31^

#### Alternative models

When the data exhibit over‐dispersion or under‐dispersion, the simple Poisson model may not be appropriate. Alternative models include the following.

##### Negative binomial regression

The negative binomial model is suitable for over‐dispersed count data. Its probability function is defined as:

(2) PrXi=xi=Γxᵢ+1αΓxᵢ+1Γ1α11+μᵢα1α11+μᵢαxi,α>0

where *α* is the dispersion parameter. In this model, the mean and variance are given by:

(3) $$E\;\left(X_{i}\right)=\mu_{i},\mathrm{Var}\;\left(x_{i}\right)=\mu_{ᵢ}\left(1+\mu ᵢ\alpha \right)$$

If *α* = 0, the negative binomial model reduces to the Poisson model.^27^, ^32^

##### Generalized Poisson regression

The generalized Poisson model can handle both over‐dispersion and under‐dispersion. Its probability function is defined as:

(4) PrXi=xi=μi1+αμixi1+αxxi−1xi!exp−μi1+αxi1+αxi

where *α* is the dispersion parameter. If *α* < 0, the data are over‐dispersed; if *α* > 0, the data are under‐dispersed. The mean and variance are:

(5) $$E\;\left(X_{i}\right)=\mu_{i},\mathrm{Var}\;\left(X_{i}\right)={\left(1+\alpha \mu_{i}\right)}^{2}\;\mu ᵢ$$

When *α* = 0, the model reduces to the standard Poisson model.^27^, ^33^, ^34^

#### Incidence rate ratio

In count models, the relationship between explanatory variables and the expected number of events is expressed through a logarithmic link function:

(6) $$\;\mathrm{xp}\;\left(X_{i}\beta \right)=\mu_{i}\to X_{i}\beta =\ln\mu_{i\;}$$

where *μ*_i_ is the expected count for unit i, *X*_i_ is the vector of explanatory variables, and *β* is the vector of regression coefficients.

The incidence rate ratio (IRR) for a variable *X*_j_ is defined as ${\mathrm{IRR}}_{j}=e^{\beta_{j.}}$

This measure indicates the proportional change in the expected count *μ* for a one‐unit increase in *X*_j_, holding other variables constant:

- if IRR_j_  > 1, an increase in *X*_j_ leads to an increase in the event rate;
- if IRR_j_  < 1, an increase in *X*_j_ leads to a decrease in the event rate.^27^, ^32^, ^34^

### Variables in the count model (dependent and independent)

The dependent variable in this study was the number of CSAPs adopted by farmers. Given its discrete and count nature, restricted to non‐negative integer values, this variable was analyzed using count regression models. The independent variables were identified based on a comprehensive literature review, semi‐structured interviews with local farmers, and consultations with agricultural and climate‐change experts. These variables capture the economic, social, institutional, and individual dimensions of farmers and are considered potential determinants of CSAP adoption.

Table 2 summarizes the independent variables employed in this study, along with their characteristics and measurement scales.

**Table 2:** Characteristics of independent variables

Independent variable	Description	Type	Categories
Human capital			
Education	Represents the respondent's level of education	Ordered	Illiterate = 1; Primary = 2; Secondary = 3; Diploma = 4; Associate = 5; Bachelor = 6; Master = 7; Doctorate = 8
Training access	Availability of specialized and technical training on climate‐smart agriculture (CSA) technology	Ordered	Strongly disagree = 1; Disagree = 2; Neutral = 3; Agree = 4; Strongly agree = 5
Social capital			
Service access	Indicates whether the respondent has access to extension services	Binary	Yes = 1; No = 0
Residence	Indicates the current place of residence of the farmer	Binary	Rural = 0; Urban = 1
Agricultural cooperative	Represents the number of agricultural cooperatives present in the respondent's place of residence	Ordered	Very low = 1; Low = 2; Middle = 3; High = 4; Very high = 5; Unknown = 6
Institutional capital			
Loan access	Indicates whether the respondent has ever obtained a loan from a bank or financial institution	Binary	Yes = 1; No = 0
Government support	Indicates the respondent's perception of the adequacy of government support for adopting CSA technology	Ordered	Strongly disagree = 1; Disagree = 2; Neutral = 3; Agree = 4; Strongly agree = 5
Crop insurance	Indicates whether respondents insure their agricultural products	Binary	Yes = 1; No = 0
Psychological capital			
Job satisfaction	Represents the respondents' level of satisfaction with their agricultural occupation	Ordered	Very low = 1; Low = 2; Middle = 3; High = 4; Very high = 5
Fertilizer trust	Represents the respondents' level of trust in using new fertilizers	Ordered	Very low = 1; Low = 2; Middle = 3; High = 4; Very high = 5
Self‐efficacy	Respondents' confidence in their ability to adopt CSA technology	Ordered	Strongly disagree = 1; Disagree = 2; Neutral = 3; Agree = 4; Strongly agree = 5

## RESULTS AND DISCUSSION

### Determinants of farmers' adoption of CSAPs

Table 3 presents the number of CSAPs adopted by farmers (the dependent variable) and summarizes the factors influencing their adoption. As Fig. 1 shows, the CSAPs were classified into four main categories: water management, agronomic practices, conservation practices, and off‐farm activities. This classification reflects the functional diversity of the practices, considering regional conditions and the types of activities performed by farmers. According to the data, the minimum number of CSAPs adopted was six, and the maximum was 22. The frequency distribution indicates that the most common adoption level was 13 practices, with 62 farmers (14.42% of respondents) falling into this category. The mean of the dependent variable was 13.6, with a variance of 10.04. The close alignment of the mean, median, and mode suggests that the data distribution is approximately symmetric and does not exhibit substantial skewness.

**Table 3:** Frequency distribution of the number of climate‐smart agriculture practices (CSAPs) adopted by farmers

Number of CSAPs	Frequency distribution		
	Number (people)	Percentage (%)	Cumulative percentage (%)
6	1	0.23	0.23
7	8	1.86	2.09
8	13	3.02	5.12
9	19	4.42	9.53
10	31	7.21	16.74
11	38	8.84	25.58
12	48	11.16	36.74
13	62	14.42	51.16
14	51	11.86	63.02
15	45	10.47	73.49
16	37	8.60	82.09
17	33	7.67	89.77
18	17	3.95	93.72
19	2	0.47	94.19
20	13	3.02	97.21
21	9	2.09	99.30
22	3	0.70	100
Total	430	100	‐

**Figure 1:**
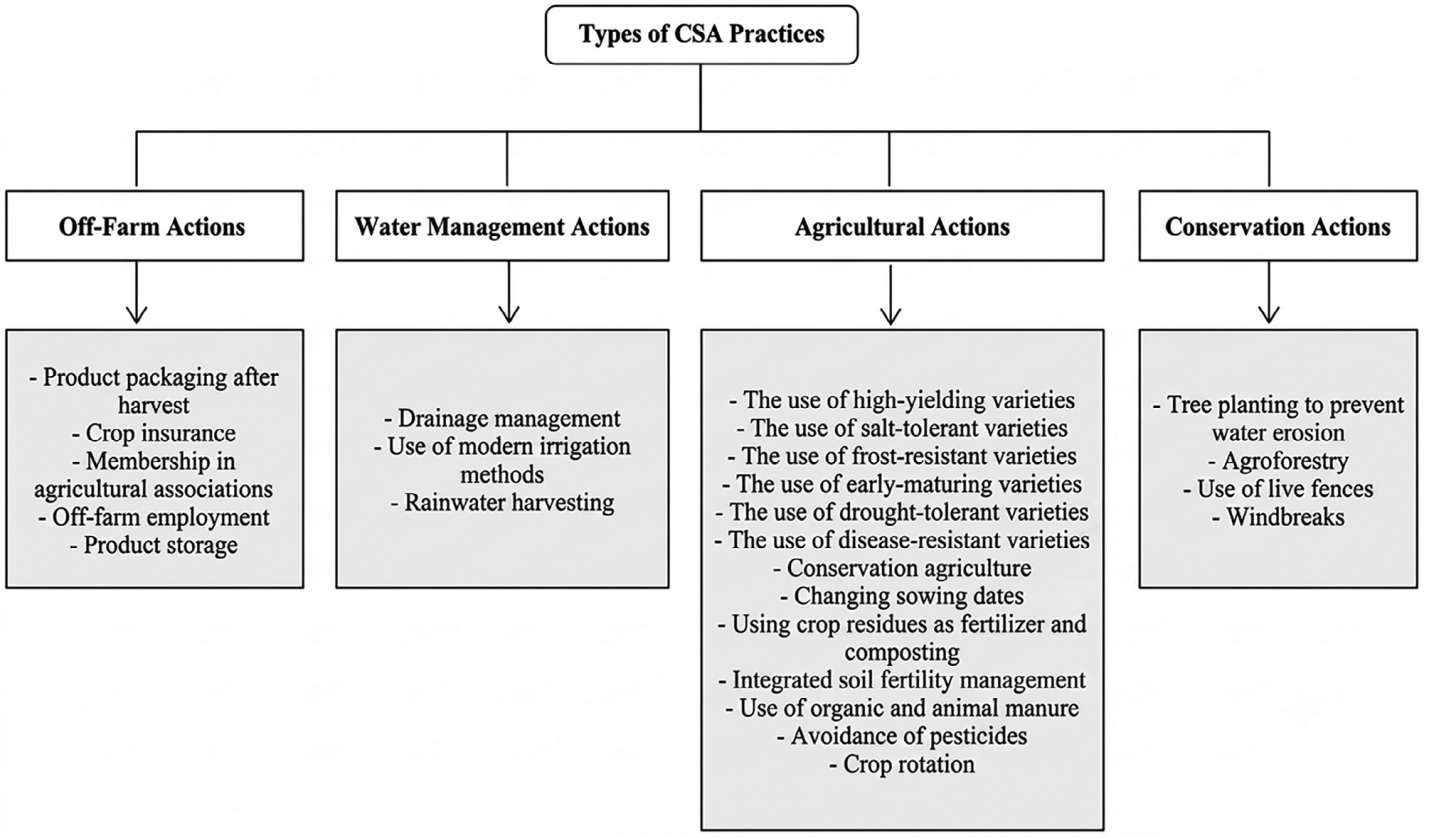
Categories of climate‐smart agriculture practices (CSAPs).

Based on these results, it can be concluded that the adoption level of CSAPs among farmers in Mashhad ranges from moderate to relatively high. On the one hand, this emphasizes the need to support farmers with low adoption levels, who require enhanced awareness and extension services. On the other hand, it highlights the importance of farmers with high adoption levels who can serve as role models and facilitate the transfer of practical knowledge to other farmers.

### Descriptive statistics

Table 4 summarizes the respondents' characteristics, providing a clear overview of the study sample. The findings indicate that over 52% of farmers have limited access to specialized training in CSA, highlighting the critical role of extension and educational services in facilitating adoption. Approximately 44% of respondents reported a high level of self‐efficacy, reflecting considerable confidence in their ability to implement new agricultural technologies.

**Table 4:** Independent variables in the count model

Variables	Group frequency (%)							
	0	1	2	3	4	5	6	7
Education		24 (5.58)	72 (16.74)	102 (23.72)	111 (25.81)	46 (10.70)	54 (12.56)	21 (4.88)
Training access		106 (24.65)	224 (52.09)	76 (17.67)	19 (4.42)	5 (1.16)		
Service access	167 (38.84)	263 (61.16)						
Residence	185 (43.02)	245 (56.98)						
Agricultural cooperative		63 (14.65)	124 (28.84)	98 (22.79)	98 (22.79)	23 (5.35)	24 (5.58)	
Loan access	315 (73.26)	115 (26.74)						
Government support		53 (12.33)	205 (47.67)	118 (27.44)	44 (10.23)	10 (2.33)		
Crop insurance	196 (45.58)	234 (54.42)						
Job satisfaction		1 (0.23)	58 (13.49)	137 (31.86)	154 (35.81)	80 (18.60)		
Fertilizer trust		0 ‐	1 (0.23)	36 (8.37)	240 (55.81)	153 (35.58)		
Self‐efficacy		2 (0.46)	73 (16.98)	113 (26.28)	190 (44.19)	52 (12.09)		

In contrast, nearly 47% of farmers rated government support for adopting CSAPs as low, and around 54% were covered by crop insurance, indicating limitations and gaps in institutional support. Most farmers reported high job satisfaction, and over 55% expressed strong trust in the use of modern fertilizers, which may positively influence the adoption of agricultural innovations. However, the data also show that more than 73% of farmers had not received any loans, highlighting financial and institutional constraints in supporting agricultural investment.

As mentioned in previous sections, one of the key assumptions of the standard Poisson model is the equality of the mean and variance of the dependent variable. Examination of the study data revealed that the mean of the dependent variable was approximately 1.35 times its variance, indicating the inefficiency of the simple Poisson model for parameter estimation. Under such circumstances, applying the standard Poisson model may lead to biased parameter estimates, unreliable standard errors, and reduced predictive accuracy.

To address this limitation and achieve a more precise analysis, a generalized Poisson model was employed. This model can accommodate both under‐dispersion and over‐dispersion in the data, providing more reliable estimates. The dispersion parameter α also reflects the degree of dispersion in the data; a negative value indicates under‐dispersion. This further indicates the suitability of the generalized Poisson model, which accurately captures the actual dispersion in the data and allows for a more precise assessment of the effects of independent variables on the count of events. Before model estimation, multicollinearity among independent variables was also examined, with a variance inflation factor of 1.05 indicating no significant multicollinearity.

Table 5 presents the estimation results of this model, including the estimated coefficients and the corresponding incidence rate ratios (IRRs = eβ). Since coefficients in generalized Poisson models are expressed on the log scale, IRRs facilitate interpretation by indicating the multiplicative change in the expected count associated with a one‐unit increase in each explanatory variable. As coefficients in Poisson models are interpreted in logarithmic terms, the hazard ratio is used to clarify the effect of each independent variable on the dependent variable, showing how a one‐unit change in an independent variable influences the expected count of the dependent variable.

**Table 5:** Factors affecting adoption of climate‐smart agricultural practices (CSAPs) (generalized Poisson model)

Variables	Coefficient	IRR	*Z*
Education	0.0187^c^	1.0188	2.79
Training access	−0.0300^b^	0.9703	−2.37
Service access	0.0619^c^	1.0639	2.91
Residence	−0.0474^b^	0.9536	−2.22
Agricultural cooperative	0.0279^c^	1.0283	3.70
Loan access	0.0861^c^	1.0900	3.71
Government support	−0.0227^b^	0.9774	−2.01
Crop insurance	0.0609^c^	1.0628	2.95
Job satisfaction	0.0269^b^	1.0273	2.46
Fertilizer trust	0.0410^b^	1.0418	2.43
Self‐efficacy	0.0348^c^	1.0354	3.14
Constant	2.1494^c^	8.5802	18.75
*α* (scattering coefficient)	−0.2873		
P‐value for the dispersion parameter (α)^a^	0.0000		

Abbreviation: IRR, incidence rate ratio.

^a^ Denotes statistical significance at the 10% significance level.

^b^ Denotes statistical significance at the 5% significance level.

^c^ Denotes statistical significance at the 1% significance level.

The negative coefficient of the *α* parameter in Table 5 indicates that the mean of the data is greater than its variance, reflecting under‐dispersion. The generalized Poisson model is therefore the most appropriate framework for analyzing these data. This model is used to estimate the coefficients and identify the factors influencing the number of climate‐smart agricultural practices adopted by farmers in Mashhad. The results of the generalized Poisson model indicate that all variables included in the model are statistically significant at various levels (1%, 5%, and 10%). The effect of each variable on the adoption of CSAPs can be interpreted based on the coefficients and IRRs.

The positive effect of education indicates that an increase in farmers' education level raised the number of CSAPs they adopted by 1.0188%. Higher education enhanced farmers' ability to comprehend and apply information about modern agricultural technologies, increasing their awareness of the benefits of CSAPs and encouraging wider adoption. Studies^35^, ^36^, ^37^, ^38^ have demonstrated that more educated farmers adopted CSAPs more rapidly, had better access to information, and were more aware of the associated benefits.

In contrast, the negative coefficient for access to workshops and technical training indicates that such access reduced the number of CSAPs adopted by 0.9703%. This may have been due to the inefficiency of training programs, mismatches between training content and farmers' actual needs, and practical barriers to applying CSA knowledge. It has been reported^39^ that, in some cases, educational content was misaligned with farmers' real needs, which lowered their willingness to adopt CSAPs. However, studies^40^, ^41^ have revealed that active participation in locally adapted training programs can positively affect CSA adoption, emphasizing the importance of high‐quality and relevant educational content.

Farmers with access to extension services were 1.0639% more likely than those without such access to adopt a greater number of CSAPs. Access to locally tailored advisory and extension services improves decision‐making and adoption rates, while also contributing to sustainable productivity and effective climate adaptation. Previous studies^9^, ^42^, ^43^, ^44^, ^45^ confirmed the positive effect of support services on CSA adoption.

The adoption of a broader set of CSAPs among farmers residing in urban areas was 0.9536% lower than among those living in rural areas. This difference was primarily due to rural households' direct dependence on natural resources, their more direct experience with climate variability, and their greater awareness of the practical benefits of these technologies, all of which collectively enhanced their motivation to adopt CSAPs. In contrast, urban farmers were less exposed to the direct impacts of climate change and thus exhibited lower motivation to adopt these practices. It was also found^46^ that, despite similar environmental attitudes, rural farmers outperform their urban counterparts in the practical adoption of CSAPs.

Membership in multiple agricultural cooperatives increases the likelihood of adopting a broader range of Climate‐Smart Agriculture (CSA) practices by 1.0283%. Cooperatives provide access to specialized knowledge, practical experience, and technical advice, and facilitate collective learning through observing successful examples. They also serve as intermediaries, providing financial and technical support and reducing operational barriers. Studies by^47^, ^48^, ^49^, ^50^, ^51^ showed that cooperatives act as effective catalysts for CSA adoption.

Farmers who had previously received loans were 1.09% more likely than those who had not to adopt more CSAPs. Loans provide the necessary financial resources for inputs, adaptation to CSAPs, and risk management, enhancing farmers' confidence and motivation. Research^52^, ^53^ confirmed the positive relationship between access to credit and CSA adoption. However, other work^54^ highlighted financial constraints and insufficient credit access as major barriers to CSA adoption, emphasizing the need to provide adequate financial resources to improve the effectiveness and uptake of these practices.

Government support was associated with a 0.9774% decrease in the number of CSAPs adopted. This negative effect likely arose from policy misalignment, inefficiencies, bureaucratic hurdles, and farmers' lack of trust in the effectiveness of programs, reducing motivation to adopt CSA innovations. The literature^54^, ^55^, ^56^, ^57^ emphasizes that the impact of government support depends on the quality, relevance, and implementation of policies.

Farmers who insured their crops were 1.0628% more likely than uninsured farmers to adopt a greater number of CSAPs. By reducing financial and psychological risks, crop insurance increased farmers' confidence and enabled more secure implementation of CSAPs. The literature^40^, ^44^, ^58^ highlights the critical role of insurance in facilitating adoption and promoting sustainable agriculture.

Greater job satisfaction motivated farmers to implement new measures and optimize production while reducing risks, increasing CSA adoption by 1.0273%. Research^59^, ^60^ has revealed the positive motivational role of job satisfaction in CSA adoption. Increased trust in new fertilizers increased the adoption of a broader range of CSAPs by 1.0418%, reflecting farmers' willingness to take risks and adapt to innovative management practices. Studies^61^, ^62^ have indicated that trust in modern technologies facilitated the adoption of smart and digital agricultural methods. Finally, farmers with greater confidence in their ability to implement CSAPs were 1.0354% more likely to adopt a greater number of CSAPs. These findings are supported by studies^63^, ^64^ that identified perceived self‐efficacy as a strong predictor of CSA adoption.

Overall, all variables examined influenced CSA adoption, with some factors facilitating it and others inhibiting it. This highlights the importance of a combination of individual, institutional, and behavioral factors in shaping the uptake of modern agricultural technologies.

## CONCLUSION

This study examined the adoption of CSAPs in Mashhad, a region with a semi‐arid climate and declining precipitation, where environmental pressures adversely affected agricultural productivity and food security. It highlighted the urgent need for resilient agricultural practices to cope with climate variability. Farmers' adoption of CSAPs is a key indicator of their capacity to adapt to climate change and their ability to manage associated risks, thereby improving resilience and sustaining agricultural production under climatic stress. Identifying the factors influencing CSA adoption is therefore important, as understanding these factors helps assess farmer preparedness, evaluate the efficacy of existing policy frameworks, isolate technological gaps, guide targeted extension training, optimize subsidy distribution, and strengthen regional agricultural infrastructure.

The study employed a generalized Poisson model, appropriate for analyzing under‐dispersed count data. The results revealed three primary determinants – access to credit, access to agricultural services, and crop insurance – representing the three main dimensions of financial capacity, technical support, and economic security in agricultural innovation. Given the importance of these factors in empowering farmers and promoting CSA adoption, the design and implementation of practical strategies and targeted policies are essential. Practical recommendations include the following.

### Enhancing financial and credit resources

Financial capacity and risk‐taking ability among farmers can be strengthened through the development of microcredit and local loan schemes tailored for rural areas. Financial institutions should be encouraged to provide green loans, offer long‐term financing at favorable interest rates, and design targeted credit packages. Such measures would facilitate critical investments in sustainable agricultural technologies, including drip irrigation and climate monitoring equipment.

### Improving technical capacity and access to services

To enhance farmers' capacity for climate change adaptation and modern technology adoption, agricultural extension networks and the field presence of local experts should be strengthened. Short‐term practical training workshops should be conducted, and online advisory services and digital information systems should be expanded. The provision of clear, user‐friendly manuals would support these developmental goals.

### Increasing economic security and reducing risks

Climate and parametric insurance schemes should be designed based on objective weather indices to reduce economic risk and facilitate confident CSA adoption. Diverse policy packages must be offered to accommodate specific regional conditions while ensuring comprehensive coverage for vulnerable farmers. Financial incentives should also be provided to encourage insurance uptake and foster innovative agricultural practices.

The findings also indicate that weak training programs and ineffective government support can hinder CSA adoption. Continuous, practical training programs focused on modern technologies and climate risk management are therefore recommended. Supportive policies should align with farmers' actual needs. They should include targeted subsidies and innovation support, and involve farmers actively in the design and implementation of training and support programs.

### Additional recommendations

#### Crop diversification and resource management

Drought‐ and salinity‐resistant crop varieties should be promoted, along with the development of local seed banks to preserve genetic diversity, reduce reliance on external inputs, and manage climate risks. Agricultural residues should be utilized for compost or bioenergy, and robust value chains should be developed to enhance resource efficiency and environmental sustainability.

#### Collaboration and cross‐sectoral networking

Partnerships among farmers, universities, research institutions, and the private sector should be facilitated. Pilot projects should be supported to enable knowledge transfer, ensure the local adaptation of CSA technologies, and permit rigorous evaluation under real conditions.

#### Development of local markets and value chains

Local markets for CSA products should be established to enhance economic incentives for sustainable practices and strengthen local food security. Regional brands should be developed and local processing capacity supported to maximize agricultural value.

#### Strengthening trust and regulatory frameworks

Trust in agricultural inputs, particularly fertilizers and other production factors, is central to CSA adoption. Strengthening this trust requires transparent monitoring, evaluation, and standardization systems for quality and safety. Integrating scientific training with farmers' field experience can deepen understanding of modern inputs and foster sustainable trust among stakeholders.

### Recommendations for future research

#### Expansion across climatic regions

Future studies should cover diverse climatic, ecological, and socio‐cultural contexts. This broader scope will clarify how environmental variability, resource availability, and cultural norms influence CSA adoption, allowing for the development of context‐specific policies and tailored interventions.

#### Development of research models and long‐term monitoring

Advanced analytical approaches, such as structural equation modeling and path analysis, can uncover causal relationships among individual, social, institutional, and environmental factors. Longitudinal studies are also essential to measure the long‐term economic and ecological impacts of CSA, including cost reduction, efficient resource use, higher farmer incomes, lower energy and water consumption, and improved soil and ecosystem health.

#### Assessment of digital technologies

Further research should evaluate the effectiveness of digital tools such as mobile applications, Internet of Things (IoT) platforms, and online advisory systems. Optimizing these technologies can improve farmers' access to climate data and strengthen decision‐making within CSA frameworks.

#### Identification of target and vulnerable groups

Recognizing vulnerable farmers – those with financial limitations or limited access to resources and training – enables the design of targeted support programs. Such initiatives can increase equity and improve the overall effectiveness of extension and capacity‐building activities.

Overall, this study shows that the effective adoption of CSA requires a holistic approach that integrates education, institutional support, market incentives, resource management, and collaborative networks. By addressing these dimensions, policymakers and practitioners can strengthen farmers' adaptive capacity, ensure agricultural resilience, and contribute to long‐term food security under changing climatic conditions.

## CONFLICT OF INTEREST

The authors declare no conflict of interest.

## FUNDING INFORMATION

This research did not receive any specific grant from funding agencies in the public, commercial, or not‐for‐profit sectors.

## Data Availability

Data will be made available on request.
